# Qualitative study of the BREATHER trial (Short Cycle antiretroviral therapy): is it acceptable to young people living with HIV?

**DOI:** 10.1136/bmjopen-2016-012934

**Published:** 2017-02-17

**Authors:** Sarah Bernays, Sara Paparini, Janet Seeley, Stella Namukwaya Kihika, Diana Gibb, Tim Rhodes

**Affiliations:** 1 Faculty of Public Health Policy, London School of Hygiene & Tropical Medicine, London, UK; 2 MRC/UVRI Uganda Research Unit on AIDS, Social Science Programme, Entebbe, Uganda; 3 MRC Clinical Trials Unit at UCL, London, UK

**Keywords:** PAEDIATRICS, HIV, antiretroviral therapy

## Abstract

**Objectives:**

A qualitative study of the BREATHER (PENTA 16) randomised clinical trial, which compared virological control of Short Cycle Therapy (SCT) (5 days on: 2 days off) with continuous efavirenz (EFV)-based antiretroviral therapy (CT) in children and young people (aged 8–24) living with HIV with viral load <50 c/mL to examine adaptation, acceptability and experience of SCT to inform intervention development.

**Setting:**

Paediatric HIV clinics in the UK (2), Ireland (1), the USA (1) and Uganda (1).

**Participants:**

All BREATHER trial participants who were over the age of 10 and aware of their HIV diagnosis were invited to participate. 49 young people from both arms of the BREATHER trial (31 females and 18 males; 40% of the total trial population in the respective sites; age range 11–24) gave additional consent to participate in the qualitative study.

**Results:**

Young people from both trial arms had initial concerns about the impact of SCT on their health and adherence, but these decreased over the early months in the trial. Young people randomised to SCT reported preference for SCT compared with CT pre-trial. Attitudes to SCT did not vary greatly by gender or country. Once short-term adaptation challenges were overcome, SCT was positively described as reducing impact of side effects, easing the pressure to carry and remember medication and enabling more weekend social activities. Young people on both arms reported frequent medication side effects and occasional missed doses that they had rarely voiced to clinical staff. Participants liked SCT by trial end but were concerned that peers who had most problems adhering could find SCT disruptive and difficult to manage.

**Conclusions:**

To realise the potential of SCT (and mitigate possible risks of longer interruptions), careful dissemination and communication post-trial is needed. SCT should be provided alongside a package of monitoring, support and education over 3 months to allow adaptation.

**Trial registration number:**

NCT 01641016

**Strengths and limitations of this study**

- Including a qualitative study in the trial has enriched our understanding of the impact and influence of Short Cycle Therapy (SCT) on young people's experiences of adherence. Understanding their perspectives and experiences is crucial for the intervention to be effective beyond the trial.
- By specifically acknowledging adherence challenges in childhood and adolescence, if framed thoughtfully, healthcare staff can use SCT to show a greater contextual understanding about the reasons why and ways in which treatment can get disrupted.
- A key limitation of our study is the narrow population on which these findings are based (relying on those agreeing to participate in the clinical trial and then also in the qualitative study).
- Being in the trial may in and of itself have been conducive to better adherence, due to the increased support and monitoring in the study, particularly in contexts with little or no routine access to this level of care.

## Introduction

Rates of antiretroviral therapy (ART) adherence tend to be lower among young people (aged 10–24) living with HIV compared with their adult counterparts in all settings, despite important geographical variations.^1–3^ Multiple social aspects of adherence for the paediatric HIV population need to be taken into consideration alongside the specific contexts of local HIV epidemics.^4^ Lack of disclosure of their HIV status and, commonly, insufficient discussions about the implications of HIV and ART often fail to adequately support young people's ongoing adherence.^5^ Limited control over their living environments and secrecy surrounding HIV and treatment-taking represent significant barriers to adherence.^6–9^ Treatment fatigue in facing a lifetime of ART is considered an important reason for poor adherence among young people living with HIV as with other long-term conditions,^10–12^ although little HIV research investigates this from the perspectives of young people themselves.

We report on the findings from a qualitative study undertaken as part of BREATHER (PENTA 16). BREATHER is a global, phase II, randomised, multicentre, non-inferiority trial testing the efficacy of Short Cycle Therapy (SCT) (5 days on /2 days off) for young people living with HIV (aged 8–24) on an efavirenz (EFV)-based combination.^13^ Among the inclusion criteria for the BREATHER trial were: having an undetectable viral load and being on an EFV-based combination for the prior 12 months. Treatment interruption interventions, including SCT, aim to encourage long-term adherence by offering patients regulated time off medication. The trial design to test having the weekends off treatment was informed by anecdotal evidence suggesting that managed interruptions can ameliorate the challenges of adhering continuously.^14^

The qualitative study aimed to explore the experiences of SCT, and of treatment and care more generally, among a sample of trial participants (aged 10–24). SCT is a behavioural intervention relying on self-administered ART and self-reported adherence. We used qualitative methods to elucidate whether SCT was an acceptable intervention to the target patient group and to inform any potential SCT roll-out, to ensure it is clinically *efficacious* and *effective* for the people involved.^15^

## Methodology

The qualitative study employed a longitudinal, mixed-methods design and took place in the UK, Ireland, the USA and Uganda. All young people recruited into the BREATHER trial in these countries, and aged 10–24, were eligible to participate in our study. This was subject to self-awareness of HIV infection (for at least 6 months), since not all trial participants were aware of their HIV-positive status. Children under the age of 10 were not deemed old enough to participate meaningfully in our qualitative research. In addition to consent procedures for the clinical trial, we carried out separate consent and assent procedures as appropriate and participants were reimbursed at rates in line with standard local research practices.

A longitudinal design was adopted. The first interview, conducted (in all three sites) towards the start of the trial explored participants' attitudes towards taking HIV treatment and whether or not this fit in with their daily lives and priorities. The second interview, conducted (in all three sites) at least 9 months into the trial, focused on their experience of being in the trial, including their attitudes towards SCT. The third interview, conducted (only in Uganda and UK) towards the end of the trial, investigated their ongoing experience of the trial and their preferences for future treatment options. We also conducted focus group discussions (FGDs) in Uganda after the trial findings had been explained to participants by clinicians in this site. The data were collected by SB, SN and two other Ugandan researchers, none of whom were known to the participants prior to the study. Interviews and FGDs lasted between 45 and 120 min and were conducted with participants in the clinic, apart from an interview conducted in one participant's home at their request. Audio-recorded data were transcribed verbatim and translated into English where appropriate. Personal identifying details have been removed.

The topic guide for each phase included uniform key area of investigation but not a list of prescribed questions. Though the overarching focus was similar, the guides were flexible enough to ensure interviewers could adapt the form and nature of the questions to the circumstances and maturity of the individual participant.

Data analysis was conducted by all members of the research team. A grounded analytic approach to qualitative thematic analysis was adopted, using systematic case comparison.^16^ ^17^ A discussion was held after each interview to consider emerging analytical ideas and opportunities to refine the interview guide and approach. The coding was done inductively and individually developed. These preliminary codes were then exchanged among the team, discussed and reconciled into an agreed coding framework, which was subsequently applied to the data. We then conducted an iterative comparison of codes extracted from the multiple interview data for each research participant. These are corroborated with the use of ‘negative case’ analysis, built by including instances in the data that differ or counteract emerging findings and explanations.

## Sample

Repeat interviews were conducted with 43 young people. The qualitative sample in each site reflects the diversity of the trial population in terms of sex, age and ethnicity. A total of 26 young people were recruited in Uganda from 1 clinic (Kampala), 7 in the UK and Ireland from 3 clinics (hospitals in London, Nottingham and Dublin) and 10 in the USA from 1 clinic (Memphis). We report combined data on the small samples in UK and Ireland to avoid participant identification.

The only difference that we noted by site was in study recruitment and engagement. In the USA, we recruited all participants who were eligible and enrolled in the trial at the time of the phase one fieldwork (numerical saturation) and in Uganda we recruited participants until we reached theoretical saturation. There were greater challenges to recruitment in the UK, for the trial and the qualitative study. While we were unable to collect data on this, our impression is of potential research fatigue given the extent of clinical trial research conducted among this relatively small clinical population. Nonetheless, overall recruitment response and retention through the repeated phases of the qualitative study was high, and the qualitative study sample also represented a significant proportion of the sample for the BREATHER trial in all countries (43/199 trial participants overall) (see tables 1 and 2).

**Table 1:** In-depth interview sample overview

Country	No.	Male	Female	On SCT	On CT	Switched or left trial	Age range at phase 1	Mean age	Response rate
Uganda	26	12	14	14	10	2 (to CT)	11–22	16	26/66
UK (and Ireland)	7	5	2	4	3	–	12–17	15	7/23
USA	10	9	1	4	5	1 (from trial)	18–22	20	10/14
Total	43	26	17	22	18	3	11–22	17	43/103

CT, continuous therapy; SCT, short cycle therapy.

**Table 2:** Focus Group Discussions sample overview

FGD	Age range at point of FGD	Mean age	No of participants	Male	Female	SCT	CT
FGD 1	13–15	13.5	6	4	2	4	2
FGD 2	15–17	15.7	7	2	5	5	2
FGD 3	19–24	21	7	4	3	2	5
FGD 4	16–20	18	5	2	3	2	3

CT, continuous therapy; FGD, focus group discussions; SCT, short cycle therapy.

We conducted four FGDs in Uganda after the trial findings had been reported to the study participants by clinicians. At this point, trial participants had moved into a follow-up phase of the trial and were continuing in their same assigned treatment arms. In addition to including a theoretically informed subsample of the interview sample, we invited six further trial participants, who had not previously been involved in the qualitative study, to take part in the FGDs to broaden our understanding of the acceptability of SCT across the trial patient group.

In this article, we have chosen to use only randomised arm and country of origin as identifiers for the quotes. This is to protect the anonymity and confidentiality of the small sample.

## Findings

We report on the experiences of participants from both arms (rather than just those on SCT) to address the question of acceptability of the intervention to the patient target group. Given that they were randomly assigned to the intervention or control arm, they all had equal chance of being put onto SCT. Hence, all participants had important insights to share about how they perceived SCT as an intervention for young people living with HIV. It is critical to note that we did not find significant differences in experience or attitudes to SCT by gender, age or country site. The slight differences we noted were limited to the style of accounting across countries and ages, but there was no variation by content.

Overall participants described a positive SCT experience and a preference for SCT over continuous therapy (CT). However, those in the SCT arm described challenges adapting to SCT in the short term. Young people from both arms discussed having initial anxieties about the impact SCT could have on their health and adherence patterns, but these concerns decreased after the first few months in the trial.

### Early concerns about SCT

In the early stages of the trial, young people from both arms discussed anxieties about the possible impact of SCT on their health and adherence patterns:

I thought that it [SCT] would be harmful … Because I was taking a break yet I was not used to that. (Uganda, SCT arm)

Some worried they might mistake or forget the days on and the days off*.* They were concerned that any mistakes would damage their health, and that they could not predict changes in their own behaviour or in their bodies:

[The thought] was bothering me just a little bit to take off two days…I might miss an extra day, because then I might be scared that something might go on and my body might change a certain way. (USA, CT arm)

### Adaptation to SCT

Concerns decreased over the first few months in the trial when participants on SCT did not observe any explicit adverse effects. However, almost all the participants on the SCT arm reported challenges in initially adapting to the new routine:

Oh, that was hectic…when I first started the study, I think the first week I think I took it on a day that I wasn't supposed to take it because I'm so used to it. But now I'm used to it. (USA, SCT arm)

Many found it difficult to deliberately miss treatment, when they had consistently been encouraged to take their pills every day:

Because being so used to taking it seven days a week and then now they're saying I can take two days off, it's like a slight change and if you don't get your mind focused…basically it's like when you're so used to something and then you're trying to change it, it takes time. (USA, SCT arm)

SCT also temporarily affected some of the participants' autonomy in treatment taking. To adapt to their new treatment schedule, some had to reverse the independence gained by managing their own treatment, and temporarily ask for supervision from their carers after having been in sole charge of their adherence for some time. The adaptation period was relatively short, and young people tended to become used to the new routine within 2–10 weeks.

Only one participant reported being unable to adapt to the changes brought by SCT and at their request was returned to CT.

It is me who even told the Doctor that I want to go out of this short cycle. Because I used to miss. If I miss I would miss even Monday. (Uganda, Changed back from SCT to CT arm)

All the others on SCT reported getting used to their new regimen and even finding that having 2 days off helped them to adhere to their medication for the remaining 5 days of the week. SCT thus worked as a reminder and as a reward:

It [SCT] gives you the courage to take your drugs daily other than in the other days. Reason being that you will say that I have missed to take the drugs in these two days that means that in the remaining days I have to be vigilant to take the drugs such that I can have the guts to rest in these other days [two days]. (Uganda, SCT arm)

Many reported finding it ‘liberating’ to not have to remember, carry and take treatment at the weekends. This enabled them to socialise more, without worrying about finding a private space to take pills:

I don't know what it is about those two days, but it's the best days ever (…) I can go somewhere and not have to worry about taking that pill. Sometimes when I take the pill, my stomach hurts sometimes (…) but I don't have to worry about that, and I don't have to worry about taking this big pill, and I don't got to worry about coming home at a certain time and taking it, I don't got to worry about getting up and taking it. I'm just free for those two days. (USA, SCT arm)

It gave me freedom inside my heart and I saw that eeh at least here I have started to be like a normal person. (Uganda, SCT arm)

It is important to note that the challenges in adapting to SCT only came up in the later waves of data collection, although many participants would have been going through these around the time of their first interview. They may have found it easier to identify a problem retrospectively once it had been addressed, but their confidence in what they could tell us in interviews also increased. Indeed, some also mentioned in later interviews that they did not voice their ambivalence or problems with SCT at the start of the trial for fear that they would be moved to the CT arm.

### Missing doses and ART side effects

SCT brought respite also because some participants felt that there was a ‘legitimate’ way to miss doses. They were reassured by the trial that their medication had continued to be effective even if they had not been adhering 100% to their regimen:

If you don't have a break you may forget to take drugs like on a Saturday or Sunday but if you are supposed to have a break it is acceptable. (Uganda, SCT arm)

SCT thus eased the pressure to *never* miss any treatment. Knowing they could have the weekends off, and worrying about missing cumulative doses, many were instead motivated to take their treatment diligently for the remainder of the week:

I'd probably have been a bit more cautious…you're already missing two days so…you kind of get the impression you can't really miss another one. (UK, SCT arm)

Nonetheless, although described by those working in the clinics as ‘exemplary adherers’ with undetectable viral loads, participants did report missing doses occasionally and intermittently—when on CT or outside of the prescribed SCT days. Participants interpreted stable viral load results as justification to avoid reporting ‘slippages’ to their clinicians:

I try not to hide anything, it's just that I probably feel like a smidge ashamed … because I'd have told them if they asked, but if they didn't ask then and I find out my viral load was undetectable then I just let out a sigh of relief and keep going. (USA, SCT arm)

Similarly, young people from both randomised arms reported frequent and sometimes disabling treatment side effects that had so far been difficult to voice in the clinic:

I used to take it while at school but I would feel dizzy. After taking it at 9:30pm I could not read by 10pm and would not be able to walk properly yet I didn't want to disturb other children so I would just lie down there. (Uganda, SCT arm)

Some participants discussed having to change the time of the day when they took their medicines, and adapt day-to-day activities to cope with side effects:

The doses at 5 … when I was in school I tried switching it to me taking it in the morning…it wasn't really such a good idea but that feeling of being high at school was not the best situation, I don't like that at all because I mean I can't concentrate, [I] feel like I'm not really there. (USA, CT arm)

Participants also reported that side effects were not felt on the days when they did not take their medication, so SCT afforded them a welcome break. In this way, some participants reported that SCT made the experience of taking treatment more bearable:

I don't get hot flushes on the weekends and I can stay up a little bit longer…It's like your body starts getting woozy and weak and now on the weekends it's like, I'm just still full of energy. So it's better, much better. (USA, SCT arm)

### Holding back ‘truths’ in the clinic

Encouragingly, most participants greatly valued their relationship with clinicians and appreciated the care and support that they received. However, this could translate into feeling under pressure to be the ‘ideal patient’ for their clinicians, which inhibited candid discussions about adherence problems.

The label of ‘exemplary adherers’ applied in the clinic to trial participants (based on their undetectable viral load) demonstrates inherent challenges within the clinical relationship: it is difficult to be informed about young people's adherence behaviour if they are so anxious about the consequences of being ‘found out’. Further, limited disclosure of non-adherence affected young people's capacity to receive tailored adherence support. Participants said it was easier to tell the researchers in our study about their missed doses because they were not connected to the clinic and did not fear that they would ‘quarrel or abuse’ them.

### Keeping the secret

We found that young people often miss doses when they are in social situations that present a risk of being seen taking their treatment. Hence participants emphasised that the many benefits of SCT stemmed from reducing the visibility of ART in social situations. This is illustrative of young people's broader concerns, which underpin non-adherence at certain times:

It becomes tiresome to take the drugs every day because there are times when you are away from home or amongst people who don't know about you…So whoever sees you becomes eager to know what you are taking or what you are hiding. In my opinion having to rest is good because sometimes you may be amongst people like on a Saturday or Sunday but you are not going to take drugs so no one will get to know about your health. (Uganda, SCT arm)

### SCT as ‘progress’ and reward

SCT, and the BREATHER trial more generally, also symbolised scientific progress to young people, a step towards a foreseeable future of better HIV therapy, when they might be able to take even less medication or a cure for HIV may be found. This may be particularly valuable for those initiated onto treatment at a very young age:

It brings hope that time will come and you stop taking even the one pill and [that one day you will] completely stop taking drugs (Uganda, CT arm)

One participant who was moved back onto the CT arm having had a spike in his/her viral load described his/her response to the trial results:

Because I have ever been there (on SCT). I know how it feels, all the happiness in it. So even though I was a little sad I still have hope that I will go back soon (to SCT). (FGD, Uganda)

### Response to the trial findings

The FGD participants in Uganda were delighted by the trial results. Many described anticipating the outcome that SCT would be ‘non-inferior’ given their own positive experiences of the intervention, but the results further endorsed their confidence in the benefits of SCT. This suggests too that there may have been greater anxiety about SCT than had been expressed during the trial:

When I heard the results, it gave me more courage to adhere to the drugs and I saw that already we had reached somewhere. We are on track. And it gave me strength and I got to know that that if it was possible then other things are coming.

We were so happy because when you get a break and something went wrong and you felt bad. You would get worried and wonder if it was because of missing. But when we heard it had worked we all became happy.

Those on the CT arm were keen to begin on SCT as soon as possible. There was, however, an understanding of the need to stay in the same arms for the duration of the trial follow-up. No one in the qualitative study on the CT arm described informally practising ‘their own SCT’ within the period of the trial. However, some FGD participants discussed being sorely tempted to switch themselves onto SCT after hearing the results. As such, they were very keen that it should be rolled out soon to those satisfying the relevant clinical criteria.

For me I would say that it is not good to make a child get used to taking milk which you will not be able to provide. You rather not and make them get used to black tea.

Despite their own positive overall experiences of SCT, many participants, from both arms, were concerned that the idea of SCT could disrupt the clarity of the adherence messages young people are given. Many of these young people would generally describe their own adherence as relatively good. Hence, they were anxious that although SCT had helped them, those who were having greater struggles with adherence would not be able to manage the structure and discipline required to adapt to the treatment interruption.

Many were thus concerned about what other positive young people might do once the trial results are made public and felt that other young people should not be told about SCT for fear of how they might apply this to their own treatment taking without supervision or monitoring: ‘They will misinterpret the study’; ‘They will say that let us also do what they are doing, yet they don't have all the facts’.

## Discussion

Findings from this qualitative study indicate that those on the SCT arm, after taking some time to adapt, expressed a preference for taking the weekends off treatment, which suggests that SCT was acceptable to them. Although preferred to CT by those in the trial, SCT may not be a viable option for everyone, because even ‘exemplary adherers’ like the young people in our study encountered initial challenges in adapting to the new routine.

The study highlighted patterns of non-disclosure of adherence behaviours common among young people living with HIV.^5^ ^18^ ^19^ However, our data have further illustrated how young people use clinical indicators to gauge whether to share information about ‘slippages’, and to justify non-disclosure of adherence issues. They interpret an undetectable viral load as a demonstration that recent missed doses are not significant. The extent and impact of medication side effects were also consistently under-reported to clinicians, and possibly within the trial. Some participants had come to perceive these side effects as inevitable and not worthy of mentioning to clinic staff. Further research with young people on EFV-based medication is urgently needed to understand how side effects might affect their adherence and their perceptions of themselves, health and HIV.^20^ ^21^

A key limitation of our study is the narrow population on which these findings are based (relying on those agreeing to participate in the clinical trial and then also in the qualitative substudy). Also, being in the trial may have been conducive to better adherence, due to the increased support and monitoring in the study, particularly in contexts with little or no routine access to this level of care. So far we have not explored the acceptability of SCT among those who refused to take part in the trial or were not eligible. It will be vital to include these young people in the future to address acceptability of SCT more broadly and robustly.

Including a qualitative study in the trial has enriched our understanding of the impact and influence of SCT on young people's experiences of adherence. This is likely to contribute to the wider success of the intervention, by informing how SCT might be rolled out outside of trial conditions. The intervention relies on young people to change the ways in which they take their treatment and they adhere to the structured 2 days interruption. Understanding their perspectives and experiences is thus crucial for the intervention to be effective beyond the trial. Despite an increasing recognition of the pertinence of qualitative research to understand pressing public health challenges, there is an ongoing reticence among many leading clinical journals to publish this research alongside trial findings.

SCT thus presents a unique opportunity to change the conversation about ART with young people. By specifically acknowledging adherence challenges in childhood and adolescence, if framed thoughtfully, healthcare staff can use SCT to show a greater contextual understanding about the reasons why and ways in which treatment can get disrupted. SCT could be used as a ‘reward’ for those who can manage to adhere well, sustaining them on the days ‘on’ ART and/or an incentive for others to put more effort into taking treatment and lowering viral load.

Yet, thoughtful planning and framing of SCT to young people is necessary for these potential benefits to be realised. As a patient-managed strategy, participants felt that SCT could pose significant risk to other young people who may independently take treatment breaks under inappropriate conditions and without assessment and monitoring. This provides further evidence of the need for careful dissemination and communication post-trial.

The initial challenges described by so many participants need to be taken into serious consideration when planning any further intervention. The adaptation period, although different for different participants, was generally only short lived but it should not be underestimated. Our findings emphasise the importance of incorporating a package of interventions to accompany any roll-out of SCT to support young people in adapting to their new routine. We would anticipate that specific support should be provided for 12–16 weeks to accompany the adaptation period for those switching to SCT and that this should be preceded by a 2–4-week preparation period of education and counselling to alleviate concerns and ensure effective understanding about the weekend break. Participants also suggested that such an intervention during this period may be further strengthened by incorporating peer support from those already on SCT. Any intervention should be subject to ongoing evaluation. Provided early adjustments are carefully managed through a tailored brief support programme, the study has shown that SCT could be successfully transformed into a welcome treatment option for young people living with HIV.
